# A system for real-time multivariate feature combination of endoscopic mitral valve simulator training data

**DOI:** 10.1007/s11548-022-02588-1

**Published:** 2022-03-16

**Authors:** Reinhard Fuchs, Karel M. Van Praet, Richard Bieck, Jörg Kempfert, David Holzhey, Markus Kofler, Michael A. Borger, Stephan Jacobs, Volkmar Falk, Thomas Neumuth

**Affiliations:** 1 Innovation Center Computer Assisted Surgery, University of Leipzig, Leipzig, Germany; 2 Department of Cardiothoracic and Vascular Surgery, German Heart Center Berlin, Berlin, Germany; 3 DZHK (German Centre for Cardiovascular Research), Partner Site Berlin, Berlin, Germany; 4 Department of Cardiovascular Surgery, Heart Center Leipzig, Leipzig, Germany; 5 Department of Cardiovascular Surgery, Charité – Universitätsmedizin Berlin, corporate member of Freie Universität Berlin, Humboldt-Universität zu Berlin, and Berlin Institute of Health, Berlin, Germany; 6 Translational Cardiovascular Technologies, Institute of Translational Medicine, Department of Health Sciences and Technology, Swiss Federal Institute of Technology (ETH) Zurich, Zurich, Switzerland

**Keywords:** Endoscopic training, Electromyography, Myo armband, Kinect, Endoscopy

## Abstract

**Purpose:**

For an in-depth analysis of the learning benefits that a stereoscopic view presents during endoscopic training, surgeons required a custom surgical evaluation system enabling simulator independent evaluation of endoscopic skills. Automated surgical skill assessment is in dire need since supervised training sessions and video analysis of recorded endoscope data are very time-consuming. This paper presents a first step towards a multimodal training evaluation system, which is not restricted to certain training setups and fixed evaluation metrics.

**Methods:**

With our system we performed data fusion of motion and muscle-action measurements during multiple endoscopic exercises. The exercises were performed by medical experts with different surgical skill levels, using either two or three-dimensional endoscopic imaging. Based on the multi-modal measurements, training features were calculated and their significance assessed by distance and variance analysis. Finally, the features were used automatic classification of the used endoscope modes.

**Results:**

During the study, 324 datasets from 12 participating volunteers were recorded, consisting of spatial information from the participants’ joint and right forearm electromyographic information. Feature significance analysis showed distinctive significance differences, with amplitude-related muscle information and velocity information from hand and wrist being among the most significant ones. The analyzed and generated classification models exceeded a correct prediction rate of used endoscope type accuracy rate of 90%.

**Conclusion:**

The results support the validity of our setup and feature calculation, while their analysis shows significant distinctions and can be used to identify the used endoscopic view mode, something not apparent when analyzing time tables of each exercise attempt. The presented work is therefore a first step toward future developments, with which multivariate feature vectors can be classified automatically in real-time to evaluate endoscopic training and track learning progress.

**Supplementary Information:**

The online version contains supplementary material available at 10.1007/s11548-022-02588-1.

## Introduction

While endoscopic surgery has many advantages over traditional open surgery in terms of blood loss, length of stay, etc. [1], the increased degree of complexity compels residents in training for cardiac surgery to dedicate their free time to training and preparation. Minimal-invasive procedures can be simulated and prepared for in mock-up operations, done with the proper endoscopic instruments on phantoms equipped with camera systems [2].

With additional image depth information adjustment to the unusual visual feedback would be shortened and the improvement of the instrument handling settle in earlier.

To evaluate the skill improvement that trainees achieve through multiple endoscopic training exercises and highlight the differences caused by the additional depth information, a system for the multivariate comparison of 2D and 3D endoscopic training was developed. Multiple studies have focused on the skill assessment by employing time-consuming scoring systems which are dependent on additional personnel, hence, this paper focuses on the development and utilization of an automated skill assessment system [3–6]. The *Simball Box* or research and development results like *TrEndo* provide skill assessment by instrument tracking, continuous attachment of instruments restrict alterations of the training setup and can interfere training through altered instrument handling [7–9]. Analyzing multiple motion analysis parameters (MAP) through instrument tracking with additional sensors or colored markers and image analysis pose smaller influences on the tools' characteristic behavior, yet, are inefficient due to instrument modification and simulator-dependent software adjustments [10–14]. Determining instrument positions and angles by edge detection alone forgoes the problem entirely, the necessary image processing increases the complexity of the system, decreases reliability in altered circumstances, and decreases portability to different phantom trainers [15–19].

Other works focus on the analysis of the training motions using motion data fusion of time-of-flight, inertial measurement, and infrared sensor data of the upper body posture as well as instrument movement [20, 21]. Furthermore, superficial electromyography (sEMG) concluded that [22] sEMG frequency shifts and decreases in activation potential can help monitor performance and skill acquisition in a meaningful quantitative way [23–26]. The combination of sEMG data with instrument tracking data was shown to be successful for surgical instrument recognition [27, 28]. Beyond skill assessment Siu et al. developed a method for automatic training optimization, tailoring exercise sessions and schedules according to skill level and desired development, to improve laparoscopic training and support medical staff during changes of operation theater, from civilian to military or vice versa [29].

In conclusion, a multivariate measurement setup, focusing on body motion and electromyography, should monitor training progress well enough, to detect and evaluate learning curve progress. The contributions of this work are the presentation of a simulator-independent system for multivariate training evaluation, processing of synchronously captured data to extract training metrics or features, and the analysis of features significances regarding temporal and endoscope-dependent differences.

## Methods

### Study design

The study was carried out at the Leipzig Heart Center and included 15 volunteering medical experts of different specializations and different levels of experience, divided into two groups. All participants were either practicing or studying a surgical profession. The corresponding ethics committee approved the presented study which complies with the Declaration of Helsinki (ethics approval number: EA2/064/19). Each participant was informed about the study’s purpose and procedure in detail. One group used the 2D endoscope and consisted of seven volunteers, while the other group employed the 3D stereoscopic endoscope mode and consisted of eight volunteers. Endoscopic exercises were performed on a fixed piece of cloth surrounded by artificial leather inside an endoscopic phantom, a simulator which had to be interacted with by hand, hence no additional robotic systems were used during this study.

**Fig. 1:**
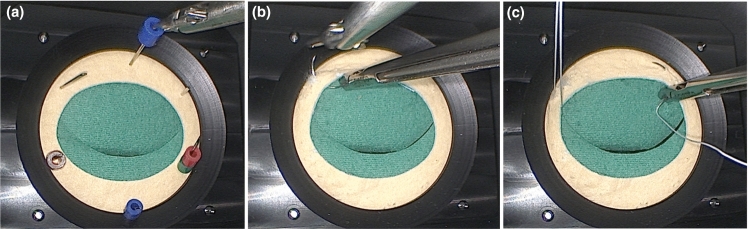
Overview of exercises performed on the phantom module during training study; **a** first exercise, bimanual carrying of 2 pegs over one lower needle each, afterwards restack one peg over one upper needle **b** second exercise, needle passing through the artificial leather of the phantom **c** needle passing with suture thread through the artificial leather and subsequent thread mounting on the outside

An endoscopic camera image of each exercise task is presented in Fig. 1, all selected tasks of this study have been validated on simulators for minimally invasive surgery before [30–33]. For the first task, participants had to use endoscopic grasping forceps and place six small plastic pegs onto six needles fixed on a circular cloth piece inside the phantom. Participants had to pick up and stack two pegs on three needles. Afterward, three plastic pegs were to be restacked onto the upper three needles. The second task was surgical needle-passing, which had to be repeated three times per attempt. To complete the attempt successfully, the needle needed to be positioned under the leather and driven through it. Afterward, it was to be passed to a needle driver in the off-hand and pulled through with a circular wrist movement. The third and final exercise required two perforations with threaded suture needles, with the addition that a thread, connected to each needle, had to be fastened in clasps outside the phantom.

The Myo armband requires an initial maximum voluntary contraction for the setup, which was performed by each participant through an initial calibration process once. The armband was not unequipped until all exercises and attempts were concluded. Each exercise attempt was initiated and concluded with a synchronizing gesture, i.e. an elevation of the main hand and arm. Exercises were repeated nine times, featuring a small break after every third attempt. For each attempt, the time to completion of the task was measured. In case the time of the exercise attempt reached 90 s, the attempt was aborted.

### Data collection

**Fig. 2:**
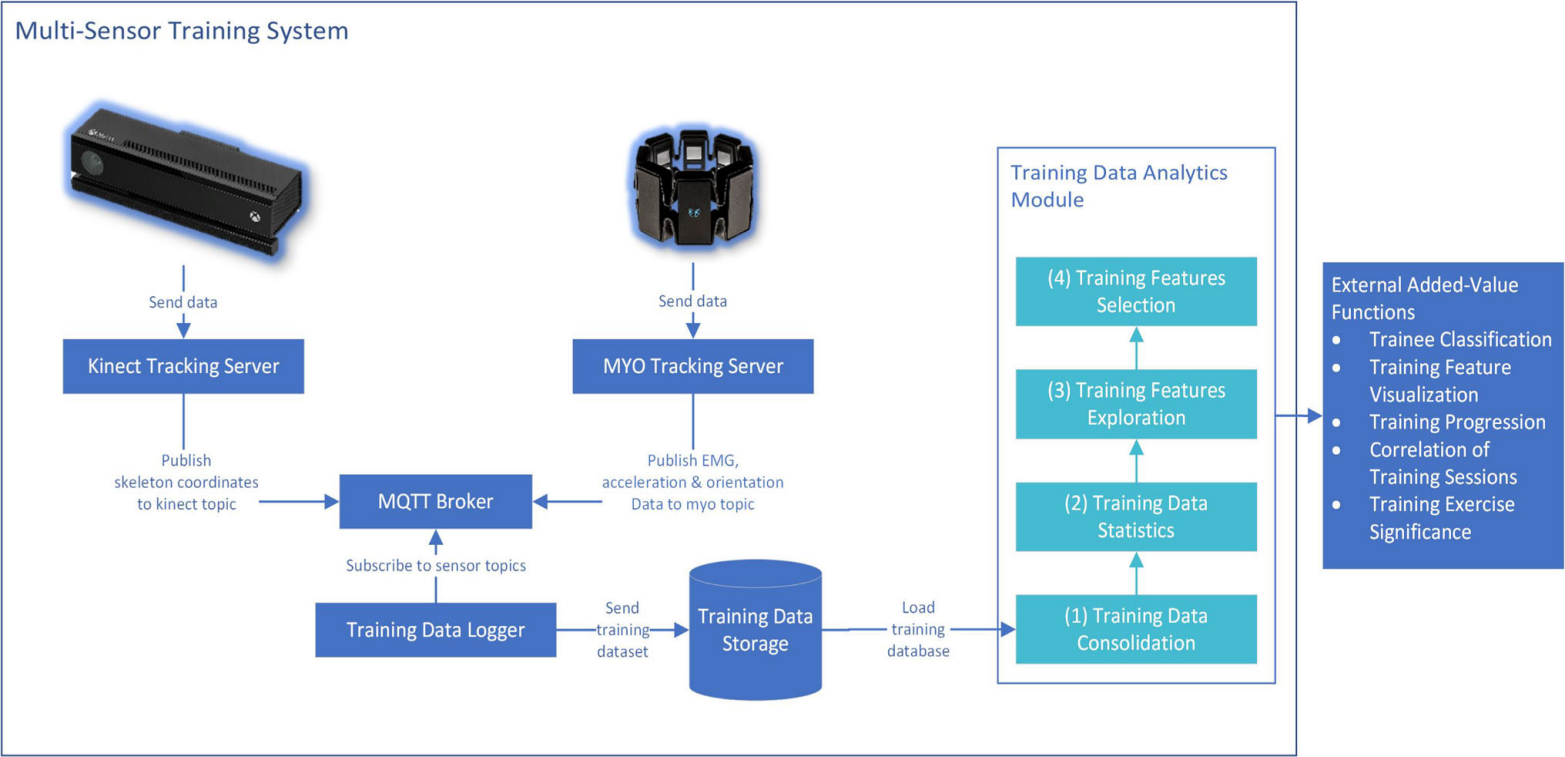
System design for multivariate laparoscopic training evaluation

Data collection was done continuously for three attempts. The authors chose a Myo armband for recording sEMG data and the Microsoft Kinect for the tracking of body and limb movement. For measurements, the Myo Gesture Control Armband was placed on the prominent bulge of the lower arm where the main muscle mass is formed [34]. For subsequent analysis, all endoscopic videos were recorded and stored as well. The devices were used in an internet of things, developed with the Message Queuing Telemetry Transport (MQTT) protocol. Device communication and data processing is summarized in Fig. 2.

### Statistical analysis

**Fig. 3:**
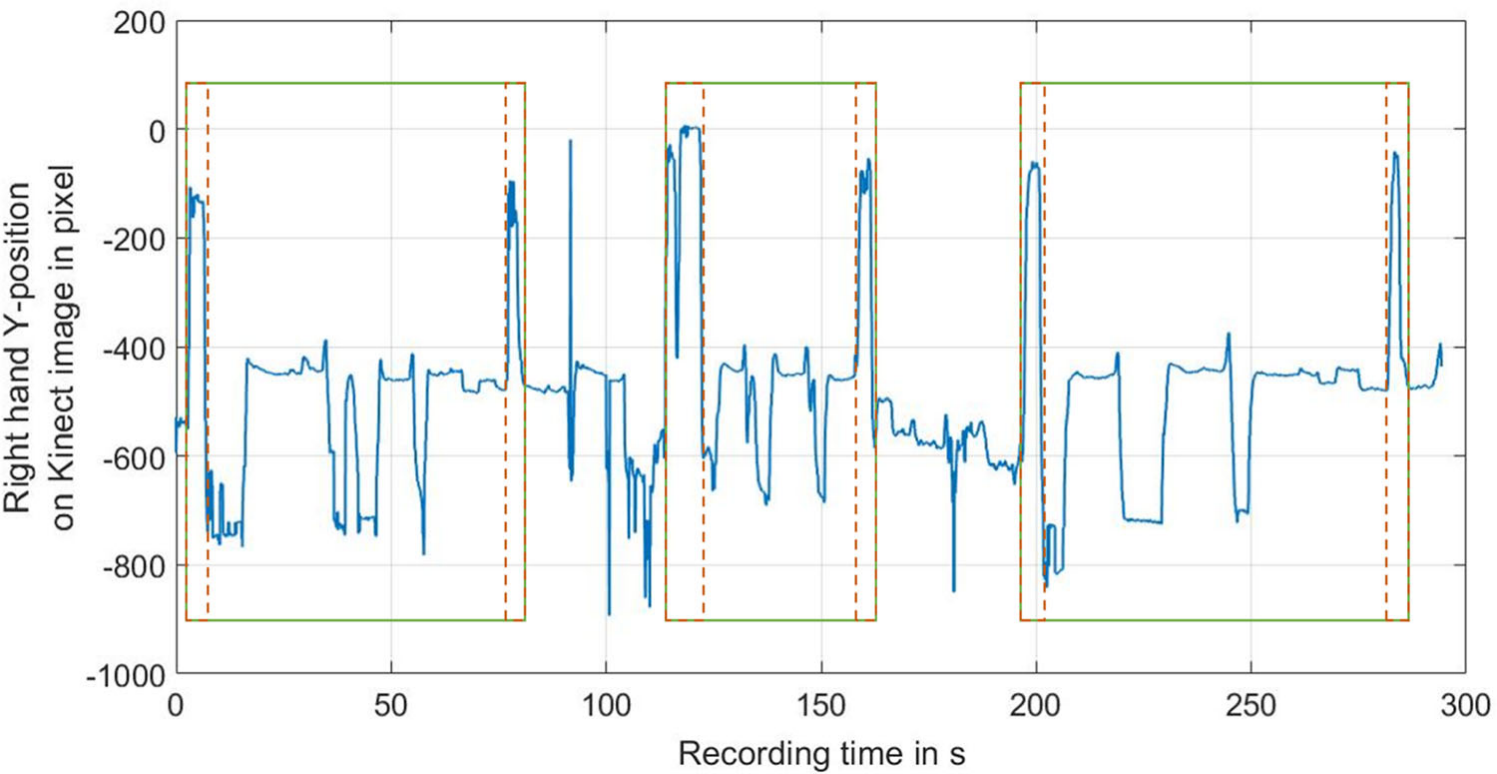
Right-Hand-Y-Position-Curve of 3 exercise attempts, recorded in one file; the curve (blue) of the right hand Y-position in the Kinect recording shows the synchronization gesture, i.e. hand raises, as an increase in the Y-position (red rectangles with dashed lines) at the beginning and end of each attempt (green rectangles with straight lines)

For data visualization and analysis, Matlab 2018b (MathWorks, Natick, USA) was employed. The gathered data of each participant was separated into nine data sequences per exercise by manually marking the points in time during which the arm raises occurred and extracting all measured data between the marked timestamps, as shown in Fig. 3. Separated Kinect and Myo measurements were used for the calculation of features, with which each exercise attempt can be represented. An overview of the chosen features with respective descriptions is presented in Table 1 with bold sEMG feature names signifying features that were averaged by the sEMG sample number of each attempt. In total, each attempt was represented by 160 different metrics. All sEMG features were calculated eight times, once for each sEMG channel, and all motion analysis parameters (MAPs) were calculated for each body part (head, spine/shoulders, left elbow, left wrist, left hand, right elbow, right wrist, right hand). After feature extraction, corrupted and incomplete data from three volunteers was excluded from further analysis.

**Table 1:** Calculated features for exercise rating per attempt

Myo armband features per sEMG channel	Description
*f*_Max_	The most powerful frequency in the filtered frequency spectrum
*f*_Min_	The least powerful frequency in the filtered frequency spectrum
*f*_Range_	The distance between the most and the least powerful frequency of the sEMG spectrum
*P*_Max_	The value of the most powerful frequency in the filtered frequency spectrum
*P*_Min_	The value of the least powerful frequency in the filtered frequency spectrum
*V*_Max_	The highest sEMG amplitude
*V*_Min_	The lowest sEMG amplitude
*V*_Range_	The difference between the highest and the lowest sEMG value
*V*_RMS_	The Root-Mean-Square over the collected sEMG values
*V*_SSC_	The number of Sign-Slope-Changes in the sEMG curve
*V*_ZC_	The number of Zero-Crossings in the sEMG curve
*V*_WFL_	The sEMG Waveform-Length
*V*_Var_	The variance of the sEMG signal
Roll_AUC_	The Area-Under-Curve of the Roll values
Pitch_AUC_	The Area-Under-Curve of the Pitch values
Yaw_AUC_	The Area-Under-Curve of the Yaw values
FES	The ratio between maximum frequency location (f_Max_) and the averaged Are-Under-Curve (AUC) of Roll, Pitch and Yaw

Kinect features per body part	Description
POC	The number of point of view changes (POC) between up and down
Trace	The distance traveled of the identified body part
Velocity	The average speed of the identified body part
Angle_Mean_	The average angle between the body axis and the normal vector of the body part
Angle_Max_	The maximum angle between the body axis and the normal vector of the body part
Angle_Min_	The minimum angle between the body axis and the normal vector of the body part
Angle_Range_	The span of the angle between the body axis and the normal vector of the body part

#### RANOVA analysis

To determine significant features for the distinction of training progress as well as possible differentiation between the two endoscope groups, a Repeated measure ANalysis Of VAriance (RANOVA) was used. The basis for model construction were the feature tables with the attempt number marking the columns and the participant numbers and their endoscope type marking the rows. The participant numbers have been omitted during the model construction. Models for repeated measurements were constructed, focusing on a sequence of attempts (1–3, 4–6, 7–9), termed session, spanning over all participants and the respective attempt numbers. The resulting models were created by combining three columns and all table row entries of one feature. Afterward, the RANOVA-p-values were calculated with epsilon correction according to Huynh–Feldt [35].

#### Feature distance calculation

For distance calculation between 2 and 3D feature results all values resulting from one kind of feature calculation were collected in one metric-specific vector per exercise and endoscope type. Afterward, the elements of each metric-specific vector with 2D values were used to calculate the median distance towards each 3D feature vector of the same exercise, resulting in 160 × 160 distance calculations per exercise. With *i* as address index for the 2D metric-specific vector ${x}_{2D}$ and *j* as address index for the 3D metric-specific vector ${x}_{3D}$, the Euclidean distance ${d}_{Eij}$ between two elements from different vectors was calculated accordingly to Eq. 1.

1 $$ d_{Eij} = \sqrt {\left( {x_{2D} \left( i \right) - x_{3D} \left( j \right)} \right)\left( {x_{2D} \left( i \right) - x_{3D} \left( j \right)} \right)^{\prime}} $$

2 $$ d_{Mij} = \sqrt {\left( {x_{2D} \left( i \right) - x_{3D} \left( j \right)} \right)C^{ - 1} \left( {x_{2D} \left( i \right) - x_{3D} \left( j \right)} \right)^{\prime}} $$

Additionally, with the calculation of the covariance matrix $C$ between the two vectors, the Mahalanobis distance $d_{Mij}$ was calculated accordingly to Eq. 2.

The distance values per comparison were accumulated in an array with ascending value order. As a result, from this comparison, the median value of the distance array was selected and stored as a representative value for the distance calculation. Furthermore, for a more efficient distance comparison, certain feature calculations were combined. To achieve this, the results of each of the six tables (two distance maps for each exercise) containing the comparison parameters, were averaged based on their affiliation which is either body part or sEMG feature. Comparison results based on sEMG values were averaged over the eight channels, resulting in one distance value per sEMG feature calculation. As for Kinect values, comparison results of each body part were averaged.

#### Classification

For the final analysis, a classification of the feature vectors for each attempt was performed, training multiple models to predict the endoscope type, which was in use during the exercise attempt of the respective feature vector. For each exercise, feature vectors were accumulated and divided into the target groups, i.e. *Ex1_2D* and *Ex1_3D* for data recorded while using either 2D or 3D endoscope during the first exercise. Before classification, all features were normalized according to the maximum and minimum overall attempts of all participants per exercise. Concluding this calculation, models for classification were trained with the classification toolbox, provided by Matlab. As a first step, each table containing the normalized features was used for the training of support vector machine (SVM), k-nearest-neighbor (KNN), decision tree models (DT), and multiple different ensemble variants.

## Results

### Study

Over two days, 15 volunteers joined the study and attempted to complete the defined tasks. The respective times of each attempt per volunteer and exercise are collected in the supplementary material document, Table SI to Table SXV.

### Data collection

Resulting from the recordings during training and the following data separation, the complete study yielded 402 datasets of different lengths, from which 81 datasets of three participants (9 attempts, 3 exercises, 3 participants) were excluded, due to transmission issues and data corruption. Further analysis procedures were executed with 160 feature calculations for overall 324 attempts (12 volunteers, 3 exercises, 9 attempts), accumulated in 160 feature tables per exercise type, having 9 columns and 12 rows.

### Statistical analysis

#### RANOVA analysis

**Fig. 4:**
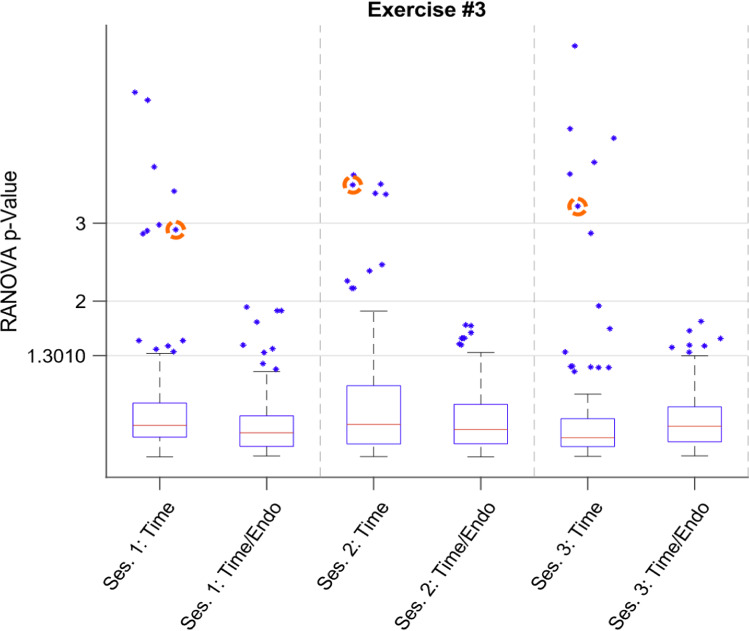
Boxplots representing RANOVA *p*-Value calculation results for each session of exercise 1, representing significance depending on time and the combination of time and the used endoscope type; outliers circled with dashed orange lines represent the reoccurring high *p*-Value of the feature *Velocity Elbow*_*L*_

The RANOVA analysis resulted in multiple p-Values describing significance in time or significance in time and between the two groups. Figure 4 shows the resulting boxplot of exercise 3, after the RANOVA calculation of each repeated model, generated from the partial feature tables. Each boxplot shows the RANOVA p-Values after epsilon correction according to Huynh and Feldt, divided into the corresponding session group the metric significance according to time-dependent evolvement as well as time and endoscope differentiation [35]. Boxplot values were converted to their negative logarithmic values (base 10) as well as three ticks on the Y-Axis, marking significance thresholds 0.05%, 0.01% and 0.001% (the ticks value being 1.3010, 2, and 3 respectively) While most whiskers reach above it, only the endoscope-dependent p-Values of session 1 (Ses. 1: Time/Endo) and the time-dependent p-Values of session 3 (Ses. 3: Time) do not feature an upper whisker above 0.05%. All boxplots show outliers far beyond 0.01% significance, with every time-dependent calculation exceeding 0.001%. As an example for repeatedly high significance, outliers belonging to the feature *Vel_Elbow_Left* have been highlighted with dashed circles.

RANOVA p-Value calculation of exercise 1 (Fig. S1) is displayed in Fig. S1 as part of the supplementary material. Only the boxplots of session 3 have whiskers above the 0.05% significance threshold, while all RANOVA calculations of the different sessions have outliers above 0.05% and 0.01% with time-dependent calculations of session 1 and session 3 feature p-Values exceeding the 0.001% threshold. Results in Fig. S2 show, that all calculations led to outliers above the 0.05% threshold and all time-dependent boxplots have outliers above the threshold marking 0.001% significance, yet none of the upper whiskers exceed 0.05%. Of the endoscope-dependent results, only the first two sessions feature outliers above 0.01%.

#### Feature distance calculation

Further significance analysis was performed through the calculation of distances between the metric-specific vectors, which resulted in 25,600 comparison values per exercise for each distance calculation algorithm. The distance calculation between metric-specific vectors resulted in six different heatmaps, consisting of the comparison results between the 2D and 3D metric-specific vectors, simplified to visualize averaged sEMG-feature-specific distances and averaged joint-specific distances.

**Fig. 5:**
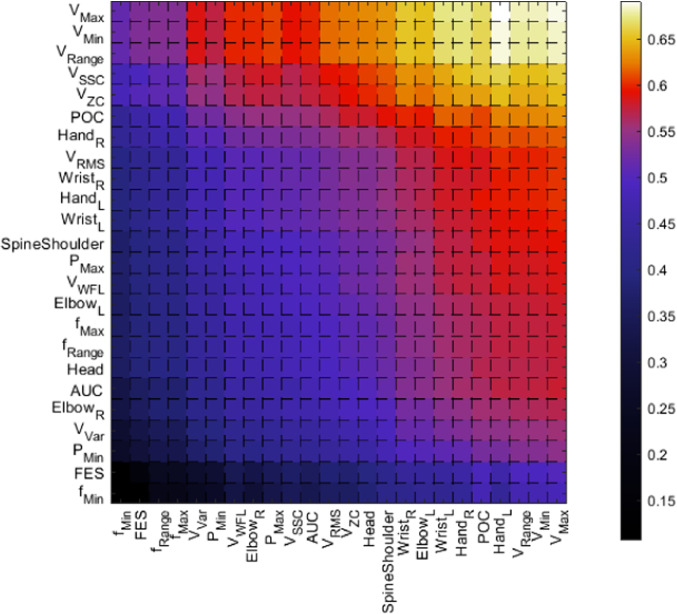
Heatmap visualizing the averaged Euclidean distances between 2D values and 3D values of the features for the exercise 3 dataset; Y-Axis contains the averaged 2D metric-specific feature vector which were used for element-wise comparison with the 3D metric-specific feature vector

Figure 5 presents the distance heatmap of exercise 3, calculated according to the Euclidean distance algorithm. All rows are ordered descending in their mean value from top to bottom, columns are ordered descending from right to left.

In Fig. 5 the features with the highest distance between 2 and 3D are *V*_Max_, followed by *V*_Min*,*_ and the subsequent difference between the two, *V*_Range_. The three features with the lowest average distance per row are, in ascending order, *f*_Min_, the *FES,* and *Elbow*_*R*_. The columns with the smallest average value are *f*_Min_, *FES* and *f*_Range_. Fig. S3, located in the supplementary materials, contains all heatmaps for visual comparison of the chosen features. As is the case with exercise 3, the plot of exercise 1 and exercise 2 show that the rows and columns with the highest mean distance are *V*_Max_, *V*_Min,_ and *V*_Range_.

**Fig. 6:**
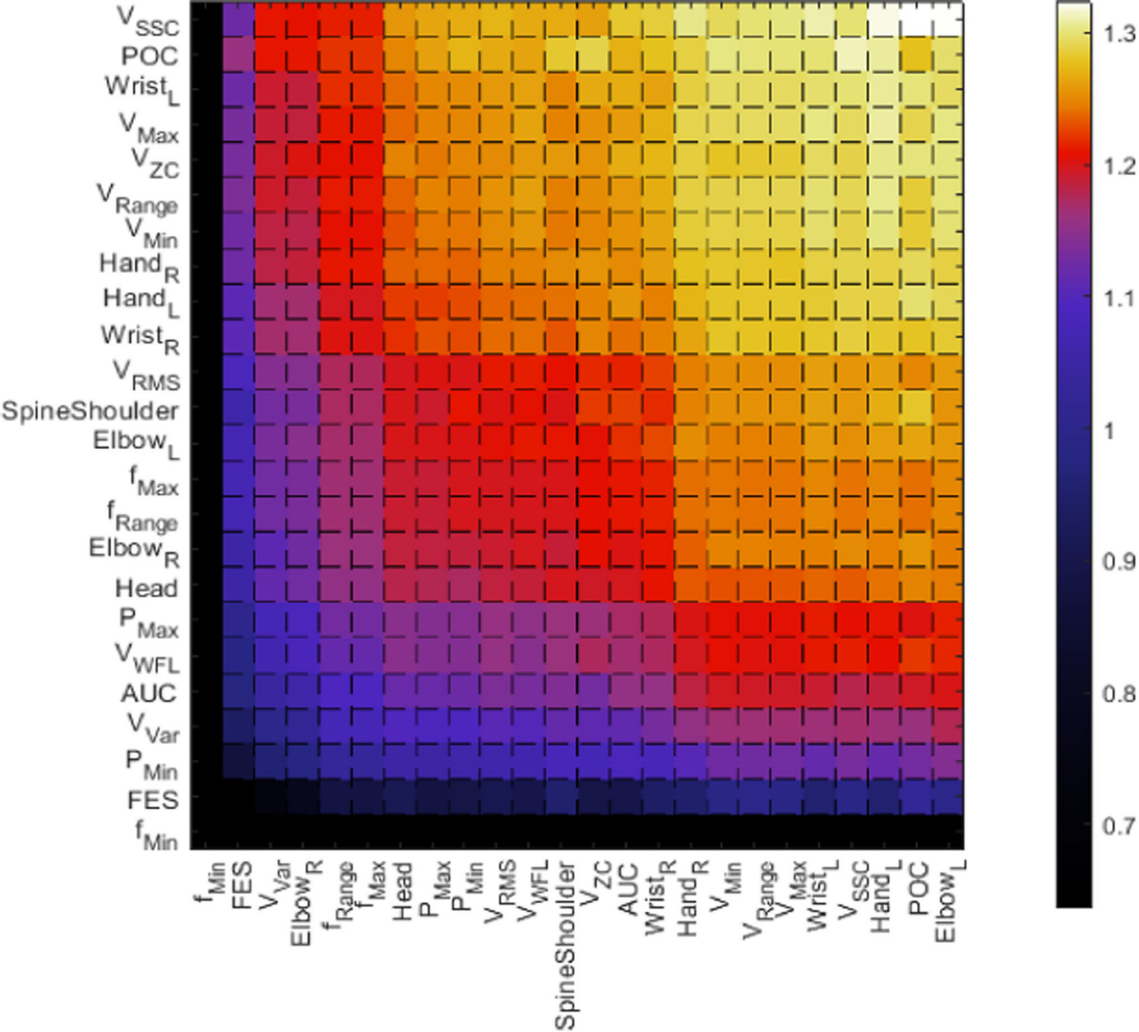
Heatmap visualizing the averaged Mahalanobis distances between 2D values and 3D values of the features for the exercise 3 dataset; Y-Axis contains the averaged 2D metric-specific feature vector which were used for element-wise comparison with the 3D metric-specific feature vector

Figure 6 is an excerpt of Fig. S4 in the supplementary material and shows median differences between each metric-specific vector, calculated according to the Mahalanobis distance. The 2D metric-specific vector with the most occurrences of high median distances is *V*_SSC_, followed by *POC* and *Wrist*_*L*_ at third place. The columns and thereby metric-specific 3D vectors with the overall highest amount of large median distances are *Elbow*_*L*_, *POC,* and *Hand*_*L*_*.* For exercise 1 in Fig. S4 the largest row values of distant comparisons are caused by the metric *POC*, *V,* and *V*_Range_, while *V*_Max_, *V*_Range,_ and *V*_Min_ are the features with the highest mean distance column-wise. Distance calculation results of Exercise 2 have a similar distribution, with *V*_ZC_, *V*_Max,_ and *V*_Range_ being at the top of the 2D distance order, while *Elbow*_*L*_*, POC*, *V*_Max_ are at the top of the 3D feature order.

#### Classification

The accuracy rates of the classification are shown in Table 2. The most left column shows the classification learner, the columns from 2nd to left until the far right shows the respective accuracy rate of each classification per exercise. Results alternate column-wise between classifications made with all available features and classifications made with only 15% of the most distant features. The highest percentage of right classifications of each column is highlighted grey. With all features, the accuracy rating for correct endoscope type prediction achieved 98.1% (exercise 1 with cubic SVM), 93.5% (exercise 2 with quadratic SVM), and 93.5% (exercise 3 with bagged DT ensemble).

**Table 2:** Accuracy rates of endoscope classification models for each exercise and the used feature selections

Classification learner	Exercise 1		Exercise 2		Exercise 3	
	All	15%	All	15%	All	15%
SVM Linear	91.7	77.8	84.3	73.1	84.3	88.9
SVM Quadratic	94.4	88.9	93.5	78.7	87.0	87.0
SVM Cubic	96.3	88.0	91.7	79.6	87.0	88.9
SVM Fine Gaussian	64.8	75.0	66.7	61.1	52.8	62.0
SVM Medium Gaussian	89.8	88.9	93.5	81.5	87.0	86.1
SVM Coarse Gaussian	83.3	71.3	79.6	73.1	80.6	85.2
KNN Fine	89.8	88.9	89.8	81.5	88.0	84.3
KNN Medium	89.8	82.4	82.4	79.6	83.3	84.3
KNN Coarse	49.1	49.1	49.1	49.1	49.1	49.1
KNN Cosine	88.0	74.1	85.2	77.8	81.5	81.5
KNN Cubic	84.3	77.8	82.4	79.6	77.8	81.5
KNN Weighted	88.9	83.3	88.0	79.6	85.2	85.2
Boosted Trees	49.1	49.1	49.1	49.1	49.1	49.1
Bagged Trees	98.1	86.1	93.5	82.4	93.5	92.6
Subspace Discriminant Ensemble	88.0	87.0	87.0	75.9	84.3	90.7
KNN Subspace	87.0	82.4	79.6	73.1	83.3	84.3
RUS Boosted Trees	57.4	49.1	56.5	52.8	49.1	57.4

Feature selection led to a feature space with 36 (exercise 1), 38 (exercise 2), and 39 (exercise 3) features. After leaving out features that do not reach the upper 15% of the distance values, the largest classification result difference was with the dataset of exercise 2 dropping by 11.1%.

The highest rate of correct predictions with a smaller feature space is, in order of exercise number, 88.9% (SVM Quadratic, Gaussian SVM Medium, Fine KNN), 82.4% (bagged DT ensemble), and 92.6% (bagged DT ensemble).

## Discussion

### Study

The results of the study were achieved over two days with multiple recording sessions, yet the yielded data is sparse. The number and especially the duration of each attempt should be increased considerably, not only to increase dataset size but also to give volunteers a larger amount of time to adjust to the task and allow for the training effect to settle more properly. The way this study was planned and executed, volunteers had little time to adjust to the task and enter a proper training mindset. Even skilled surgeons needed time to adjust to the exercises, a problem partly caused by the nature of the tasks being more relevant to beginner surgeons than already trained professionals accustomed to more complex methods.

### Data collection

The system provided the considerable advantage of synchronizing all data in real-time automatically and during recording. The data loss that occurred during this study was largely due to communication problems between the devices and the MQTT broker, a problem that needs to be addressed through additional safety measures and more development time.

### Statistical analysis

#### RANOVA analysis

The results of the RANOVA analysis show that some features exceed the chosen threshold and can be considered to possess a high significance. This supports the hypothesis that the proposed system and certain calculated features can be used as means to represent and analyze the learning progress during endoscopic training. Additionally, the results can be used to mark a difference between the use of 2D and 3D endoscopic view, however, looking at the measured times throughout the recorded tables (Table SI to Table SXV), the proposed progress does not reflect well in the actual time records, which might be attributed to the short exercise time and the little number of task attempts.

Analyzing the extracted, significant features and the RANOVA-p-Value trends of every exercise, we conclude that some features have a rising and falling significance, while some exceed the threshold during every session. Figure 4 shows the reoccurring significance of *Velocity of the left Elbow*, and the statistical impact its’ changes have on the progress during every session of exercise 3. As a continuous outlier of the time-dependent RANOVA, with all time-dependent p-Values under 0.01%, it can be safely assumed, that the feature is useful for the analysis of training progress with the proposed setup and the used exercises, at least in the early stages. Similar significant features exist, yet their time- and endoscope-dependent significance rise above and fall below the different significance thresholds. This may be attributed to the learning process as well, causing former significant features to lose informational value once the trainee reached a skill level. With the feature only being of importance during the first few tries and ceasing to visualize made progress once a certain degree of competence has been reached, the acquisition of a certain skill level can be marked with the irrelevance of the feature or, vice versa, the increase of significance in features with no former informational value. Dependent on what features show significant behavior during the training, it could be concluded what kind of skill level the trainee possesses at the start of the training session, how it changes during the session, and that a feature space is justified. This could allow for the interpretation of how well a trainee progressed throughout an overall training schedule, comparing session results, and allow for a qualified assessment of the usefulness of exercises, similar to Siu et al. [29].

#### Feature distance calculation

The results of the median calculation for the distances show, that the system can be used to evaluate combined data and distinguish between 2D-endoscopic and 3D-endoscopic vision during endoscopic training. The repeatedly high distances of amplitude describing sEMG features like *V*_Max_ and the continuously prominent *V*_ZC_ in Fig. S3 prove their significance when differentiating between the two endoscope types. The calculation algorithms result in different distance distributions, with the heatmaps based on Euclidean distance calculation showing more prominent gradients between the highest and the medium distances, noticeable at the border between *V*_Range_ and *V*_SSC_ at the y-axis of Fig. 5. Features with high distances are very distinctive and an analysis of the top 15% distance comparison reveals, that no distance map of Sig. S3 has more than 1/5th of the comparisons reaching the 85% of the maximum distance. This emphasizes their influence in the differentiation between the two endoscopic view modes (2D and 3D endoscope), that while not apparent in the time records, seem to have an influence on sEMG and some motion-related metrics.

The distance distributions in the heatmaps in Fig. 6 and Fig. S4 are more uniform with decreased gradients between ordered comparison results. While favorites for analyzing data between and the prediction of the endoscopic types are not as distinctive as they were in the Euclidean distance maps, reoccurring high distance values for amplitude*-*related sEMG features emphasize their significance and the relevance of the different muscle activation amplitudes during the training session.

#### Classification

The classification results proved, that there are distinctive differences between the endoscopic uses, which reflect in the measured motion- and muscle-related data. The high values of right predictions among the different classification learners support the claim, that the proposed setup and methods enable an endoscopic training analysis, which can also provide analysis results that do not reflect in simple time measurements.

The highest rate of correct predictions was achieved with the dataset of exercise 1, leading to the conclusion, that the difference in endoscope use is more apparent in exercise 1 than in exercises 2 and 3. This might be due to the fact, that exercises 2 and 3 resemble parts of actual surgical techniques and provide familiar actions. Exercise 1 is more abstract with nine depth-based stacking tasks instead of maximal 3 needle passing procedures, making the effects of the improved view provided by the 3D endoscope more apparent. Another factor for the decrease in the possible distinction between 2 and 3D, occurring in the later exercises, could be the learning effect, through which the trainees also grow more accustomed to the endoscopic view and the laparoscopic exercise. Volunteers using the 2D endoscope would struggle less after their first attempts at laparoscopic training during the first task and the initial benefit of the stereoscopic view would decrease. It can be argued that the trainees already made small progress in the learning curve, had a better sense for the instrument positions, required less focus on their depth approximation in the 2D image, and approached efficiency they would have had when provided with the stereoscopic view.

## Conclusion

The work presented in this paper focused on the analysis of data acquired with a multimodal device setup. The results largely support the claim, that the chosen approach and the used setup are well-suited to identify and emphasize progress in a trainee’s surgical skill, familiarity with the exercise, and conscious as well as subconscious control over the endoscopic instrument. The proposed device combination is a basis for a system, usable for the evaluation of the learning progress during endoscopic surgery training at any desired trainer. Analysis of the multimodal data enabled the identification of features well suited for the differentiation between data recorded during 2D endoscope and data recorded during 3D endoscope training. A proof-of-concept classification with classification learners resulted in accuracy results reaching up to 98.1% for 2D/3D classification. Leaving out features, following significance analysis results, the highest achieved classification was 92.6%. In conclusion, results from the training measurements and the classification of the calculated features support the claim, that the automatic, multimodal observation and evaluation of endoscopic training with the proposed setup is valid.

Yet the initial work is inconclusive, especially regarding the evaluation of actual learning progress, largely due to the limited size of the training data. The attempts per exercise were too few with not enough time per attempt. The next steps are improvement of communication stability, enabling real-time feature analysis, and conducting a study with more exercise attempts, larger time frames, and more volunteers.

## Supplementary Information

Below is the link to the electronic supplementary material.

Supplementary file1 (DOCX 383 kb)

## Data Availability

The datasets generated and analyzed during the current study are available from the corresponding author on reasonable request.

## Code availability

The entire code for the system setup, feature calculations, and subsequent analysis is available from the corresponding author on reasonable request.
