# Supplementary material for: A system for real-time multivariate feature combination of endoscopic mitral valve simulator training data
Source: Int J Comput Assist Radiol Surg. 2022 Mar 16;17(9):1619–31. doi: 10.1007/s11548-022-02588-1 (PMC9463288; doi:10.1007/s11548-022-02588-1)
Supplement: Supplementary file 1 — Supplementary file1 (DOCX 383 kb) [file 11548_2022_2588_MOESM1_ESM.docx]

^[[1]](#endnote-1)^

**Supplementary Material**

Table SI
Exercise Time Overview for Proband 1 with 2D-Endoscope

| **Session Number** | **Attempt Number** |  | **Exercise Number** |  |
| --- | --- | --- | --- | --- |
|  |  | **1** | **2** | **3** |
| **1** | **1** | 01:10 | 01:00 | 01:30 |
| **1** | **2** | 01:00 | 01:30 | 01:30 |
| **1** | **3** | 01:30 | 01:30 | 00:55 |
| **2** | **4** | 01:10 | 01:30 | 00:56 |
| **2** | **5** | 00:47 | 01:30 | 00:56 |
| **2** | **6** | 01:13 | 01:30 | 01:02 |
| **3** | **7** | 01:30 | 00:59 | 01:12 |
| **3** | **8** | 00:59 | 01:30 | 01:17 |
| **3** | **9** | 01:09 | 00:40 | 00:49 |

Table SII
Exercise Time Overview for Proband 2 with 2D-Endoscope

| **Session Number** | **Attempt Number** |  | **Exercise Number** |  |
| --- | --- | --- | --- | --- |
|  |  | **1** | **2** | **3** |
| **1** | **1** | 01:30 | 00:27 | 01:28 |
| **1** | **2** | 01:30 | 01:30 | 01:13 |
| **1** | **3** | 01:30 | 01:30 | 01:30 |
| **2** | **4** | 01:30 | 01:26 | 01:30 |
| **2** | **5** | 01:30 | 00:36 | 01:03 |
| **2** | **6** | 01:30 | 01:28 | 01:04 |
| **3** | **7** | 01:28 | 01:30 | 00:50 |
| **3** | **8** | 01:30 | 00:41 | 01:09 |
| **3** | **9** | 01:20 | 01:30 | 00:59 |

Table SIII
Exercise Time Overview for Proband 3 with 2D-Endoscope

| **Session Number** | **Attempt Number** |  | **Exercise Number** |  |
| --- | --- | --- | --- | --- |
|  |  | **1** | **2** | **3** |
| **1** | **1** | 01:18 | 01:30 | 01:30 |
| **1** | **2** | 01:30 | 01:30 | 01:30 |
| **1** | **3** | 01:30 | 00:50 | 01:17 |
| **2** | **4** | 01:17 | 01:30 | 01:30 |
| **2** | **5** | 01:05 | 00:53 | 01:30 |
| **2** | **6** | 01:00 | 01:30 | 01:17 |
| **3** | **7** | 01:01 | 00:49 | 01:30 |
| **3** | **8** | 00:36 | 01:30 | 00:59 |
| **3** | **9** | 00:57 | 01:30 | 00:55 |

Table SIV
Exercise Time Overview for Proband 4 with 2D-Endoscope

| **Session Number** | **Attempt Number** |  | **Exercise Number** |  |
| --- | --- | --- | --- | --- |
|  |  | **1** | **2** | **3** |
| **1** | **1** | 00:50 | 01:30 | 01:30 |
| **1** | **2** | 01:30 | 01:30 | 01:30 |
| **1** | **3** | 01:30 | 01:30 | 01:30 |
| **2** | **4** | 01:30 | 01:30 | 01:12 |
| **2** | **5** | 01:30 | 01:18 | 00:56 |
| **2** | **6** | 01:30 | 01:30 | 01:26 |
| **3** | **7** | 01:14 | 00:40 | 01:30 |
| **3** | **8** | 01:02 | 01:30 | 01:10 |
| **3** | **9** | 01:09 | 00:41 | 01:09 |

Table SV
Exercise Time Overview for Proband 5 with 2D-Endoscope

| **Session Number** | **Attempt Number** |  | **Exercise Number** |  |
| --- | --- | --- | --- | --- |
|  |  | **1** | **2** | **3** |
| **1** | **1** | 01:30 | 01:30 | 00:55 |
| **1** | **2** | 01:23 | 01:30 | 00:30 |
| **1** | **3** | 01:03 | 01:15 | 00:36 |
| **2** | **4** | 00:57 | 01:27 | 00:37 |
| **2** | **5** | 00:58 | 00:59 | 00:33 |
| **2** | **6** | 00:57 | 00:43 | 00:54 |
| **3** | **7** | 01:23 | 00:39 | 00:27 |
| **3** | **8** | 01:12 | 00:46 | 00:27 |
| **3** | **9** | 00:55 | 01:10 | 00:32 |

Table SVI
Exercise Time Overview for Proband 6 with 2D-Endoscope

| **Session Number** | **Attempt Number** |  | **Exercise Number** |  |
| --- | --- | --- | --- | --- |
|  |  | **1** | **2** | **3** |
| **1** | **1** | 00:47 | 01:25 | 00:38 |
| **1** | **2** | 00:40 | 00:48 | 00:34 |
| **1** | **3** | 00:54 | 01:03 | 00:31 |
| **2** | **4** | 00:48 | 00:52 | 00:35 |
| **2** | **5** | 00:43 | 00:55 | 00:29 |
| **2** | **6** | 00:53 | 01:06 | 00:28 |
| **3** | **7** | 00:43 | 01:12 | 00:34 |
| **3** | **8** | 01:22 | 01:15 | 00:35 |
| **3** | **9** | 00:46 | 00:43 | 00:29 |

Table SVII
Exercise Time Overview for Proband 7 with 2D-Endoscope

| **Session Number** | **Attempt Number** |  | **Exercise Number** |  |
| --- | --- | --- | --- | --- |
|  |  | **1** | **2** | **3** |
| **1** | **1** | 01:30 | 01:30 | 00:47 |
| **1** | **2** | 01:22 | 00:40 | 00:38 |
| **1** | **3** | 01:30 | 01:06 | 00:32 |
| **2** | **4** | 01:30 | 00:52 | 00:32 |
| **2** | **5** | 01:07 | 00:58 | 00:29 |
| **2** | **6** | 01:14 | 00:51 | 00:29 |
| **3** | **7** | 01:30 | 00:55 | 00:29 |
| **3** | **8** | 01:19 | 00:53 | 00:32 |
| **3** | **9** | 01:17 | 01:13 | 00:30 |

Table SVIII
Exercise Time Overview for Proband 1 with 3D-Endoscope

| **Session Number** | **Attempt Number** |  | **Exercise Number** |  |
| --- | --- | --- | --- | --- |
|  |  | **1** | **2** | **3** |
| **1** | **1** | 01:27 | 01:30 | 01:30 |
| **1** | **2** | 01:15 | 01:09 | 01:17 |
| **1** | **3** | 00:54 | 01:30 | 01:30 |
| **2** | **4** | 01:30 | 01:30 | 01:30 |
| **2** | **5** | 01:15 | 00:41 | 01:13 |
| **2** | **6** | 01:24 | 01:30 | 01:30 |
| **3** | **7** | 01:08 | 01:18 | 01:07 |
| **3** | **8** | 01:30 | 01:30 | 00:54 |
| **3** | **9** | 01:29 | 01:30 | 01:30 |

Table SIX
Exercise Time Overview for Proband 2 with 3D-Endoscope

| **Session Number** | **Attempt Number** |  | **Exercise Number** |  |
| --- | --- | --- | --- | --- |
|  |  | **1** | **2** | **3** |
| **1** | **1** | 01:30 | 01:30 | 01:12 |
| **1** | **2** | 01:05 | 01:30 | 01:03 |
| **1** | **3** | 01:30 | 00:27 | 01:07 |
| **2** | **4** | 01:14 | 01:30 | 01:03 |
| **2** | **5** | 00:59 | 01:08 | 00:57 |
| **2** | **6** | 00:57 | 01:29 | 00:53 |
| **3** | **7** | 01:14 | 01:01 | 00:50 |
| **3** | **8** | 01:13 | 01:03 | 00:49 |
| **3** | **9** | 00:56 | 01:24 | 00:59 |

Table SX
Exercise Time Overview for Proband 3 with 3D-Endoscope

| **Session Number** | **Attempt Number** |  | **Exercise Number** |  |
| --- | --- | --- | --- | --- |
|  |  | **1** | **2** | **3** |
| **1** | **1** | 01:25 | 01:26 | 01:01 |
| **1** | **2** | 01:30 | 01:01 | 00:36 |
| **1** | **3** | 01:05 | 01:15 | 00:43 |
| **2** | **4** | 00:50 | 00:58 | 01:30 |
| **2** | **5** | 00:52 | 00:44 | 00:35 |
| **2** | **6** | 00:56 | 00:48 | 01:07 |
| **3** | **7** | 01:25 | 01:06 | 00:37 |
| **3** | **8** | 00:56 | 01:21 | 00:37 |
| **3** | **9** | 00:54 | 00:42 | 00:32 |

Table SXI
Exercise Time Overview for Proband 4 with 3D-Endoscope

| **Session Number** | **Attempt Number** |  | **Exercise Number** |  |
| --- | --- | --- | --- | --- |
|  |  | **1** | **2** | **3** |
| **1** | **1** | 01:30 | 01:30 | 01:30 |
| **1** | **2** | 01:30 | 01:30 | 01:30 |
| **1** | **3** | 01:20 | 01:30 | 01:30 |
| **2** | **4** | 01:30 | 01:30 | 01:30 |
| **2** | **5** | 01:30 | 00:47 | 01:30 |
| **2** | **6** | 01:28 | 01:30 | 01:30 |
| **3** | **7** | 01:15 | 01:30 | 01:12 |
| **3** | **8** | 01:17 | 01:30 | 01:16 |
| **3** | **9** | 01:11 | 00:49 | 01:25 |

Table SXII
Exercise Time Overview for Proband 5 with 3D-Endoscope

| **Session Number** | **Attempt Number** |  | **Exercise Number** |  |
| --- | --- | --- | --- | --- |
|  |  | **1** | **2** | **3** |
| **1** | **1** | 00:50 | 00:45 | 00:30 |
| **1** | **2** | 00:52 | 00:44 | 00:34 |
| **1** | **3** | 00:45 | 01:10 | 00:43 |
| **2** | **4** | 00:41 | 00:38 | 00:24 |
| **2** | **5** | 00:44 | 00:33 | 00:26 |
| **2** | **6** | 00:55 | 00:26 | 00:28 |
| **3** | **7** | 00:36 | 01:03 | 00:24 |
| **3** | **8** | 00:41 | 00:34 | 00:31 |
| **3** | **9** | 00:38 | 00:38 | 00:26 |

Table SXIII
Exercise Time Overview for Proband 6 with 3D-Endoscope

| **Session Number** | **Attempt Number** |  | **Exercise Number** |  |
| --- | --- | --- | --- | --- |
|  |  | **1** | **2** | **3** |
| **1** | **1** | 00:50 | 00:55 | 00:32 |
| **1** | **2** | 00:37 | 00:48 | 00:34 |
| **1** | **3** | 00:35 | 00:55 | 00:31 |
| **2** | **4** | 00:40 | 00:41 | 00:33 |
| **2** | **5** | 00:40 | 01:05 | 00:35 |
| **2** | **6** | 00:37 | 00:49 | 00:42 |
| **3** | **7** | 00:51 | 00:45 | 00:35 |
| **3** | **8** | 00:37 | 00:40 | 00:29 |
| **3** | **9** | 00:32 | 00:40 | 00:25 |

Table SXIV
Exercise Time Overview for Proband 7 with 3D-Endoscope

| **Session Number** | **Attempt Number** |  | **Exercise Number** |  |
| --- | --- | --- | --- | --- |
|  |  | **1** | **2** | **3** |
| **1** | **1** | 00:51 | 01:20 | 00:32 |
| **1** | **2** | 00:56 | 00:38 | 00:32 |
| **1** | **3** | 00:53 | 00:45 | 00:23 |
| **2** | **4** | 00:42 | 00:35 | 00:25 |
| **2** | **5** | 00:49 | 00:52 | 00:26 |
| **2** | **6** | 00:54 | 00:54 | 00:16 |
| **3** | **7** | 00:37 | 00:35 | 00:21 |
| **3** | **8** | 00:45 | 00:41 | 00:20 |
| **3** | **9** | 00:35 | 00:29 | 00:20 |

Table SXV
Exercise Time Overview for Proband 8 with 3D-Endoscope

| **Session Number** | **Attempt Number** |  | **Exercise Number** |  |
| --- | --- | --- | --- | --- |
|  |  | **1** | **2** | **3** |
| **1** | **1** | 01:24 | 01:25 | 00:45 |
| **1** | **2** | 01:04 | 00:45 | 00:37 |
| **1** | **3** | 01:11 | 01:02 | 00:36 |
| **2** | **4** | 01:10 | 00:51 | 00:33 |
| **2** | **5** | 01:04 | 00:44 | 00:44 |
| **2** | **6** | 01:28 | 00:52 | 00:27 |
| **3** | **7** | 01:01 | 00:46 | 00:30 |
| **3** | **8** | 00:54 | 00:36 | 01:30 |
| **3** | **9** | 00:57 | 00:31 | 00:34 |


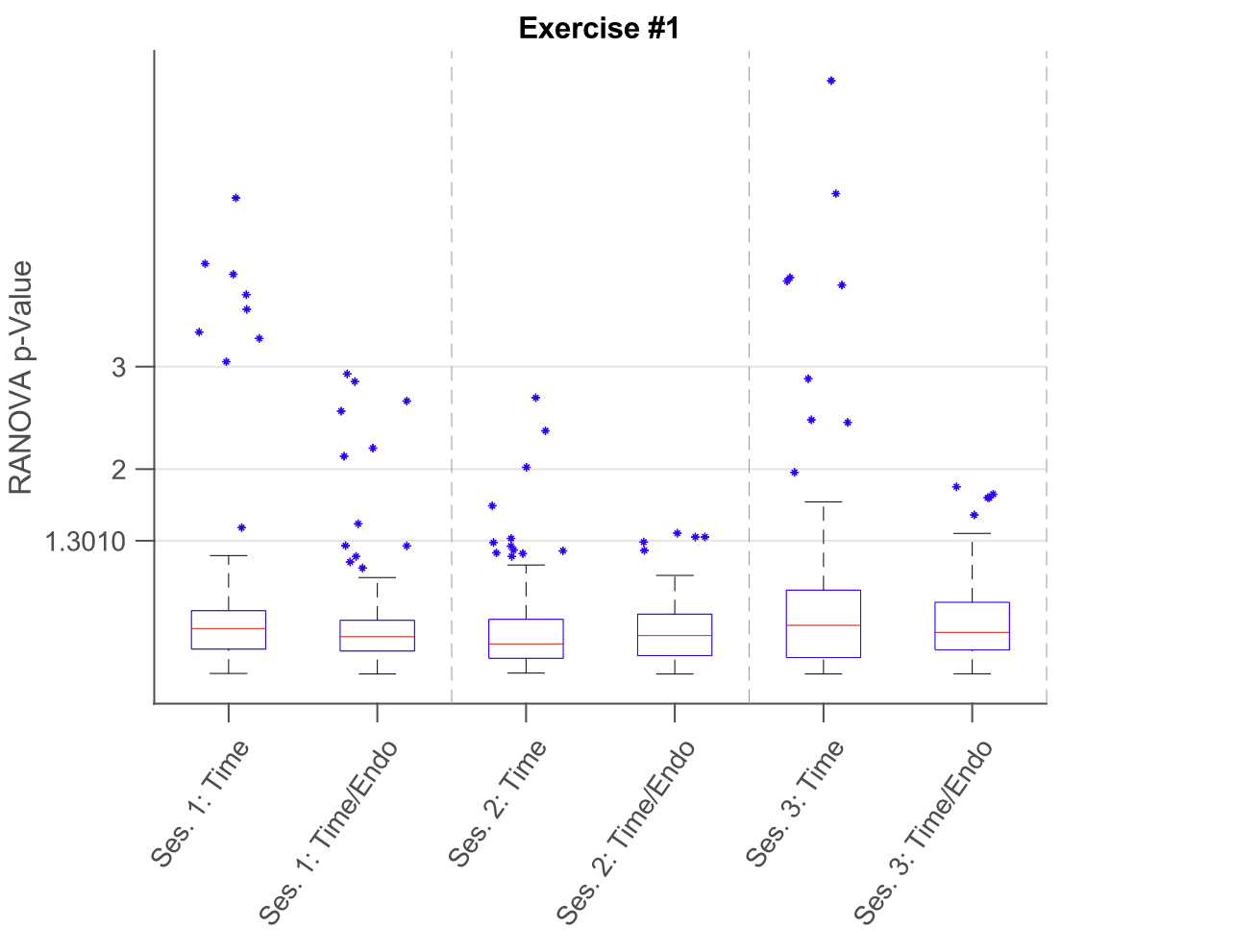


Fig. S1. Boxplots representing RANOVA p-Value calculation results for each session of exercise 1 converted to negative logarithmic (base 10) counter-part values, representing significance depending on time and the combination of time and the used endoscope type; ticks of the Y-axis mark the p-Value threshholds 0.05%, 0.01% and 0.001% (converted values being 1.3010, 2 and 3 respectively)


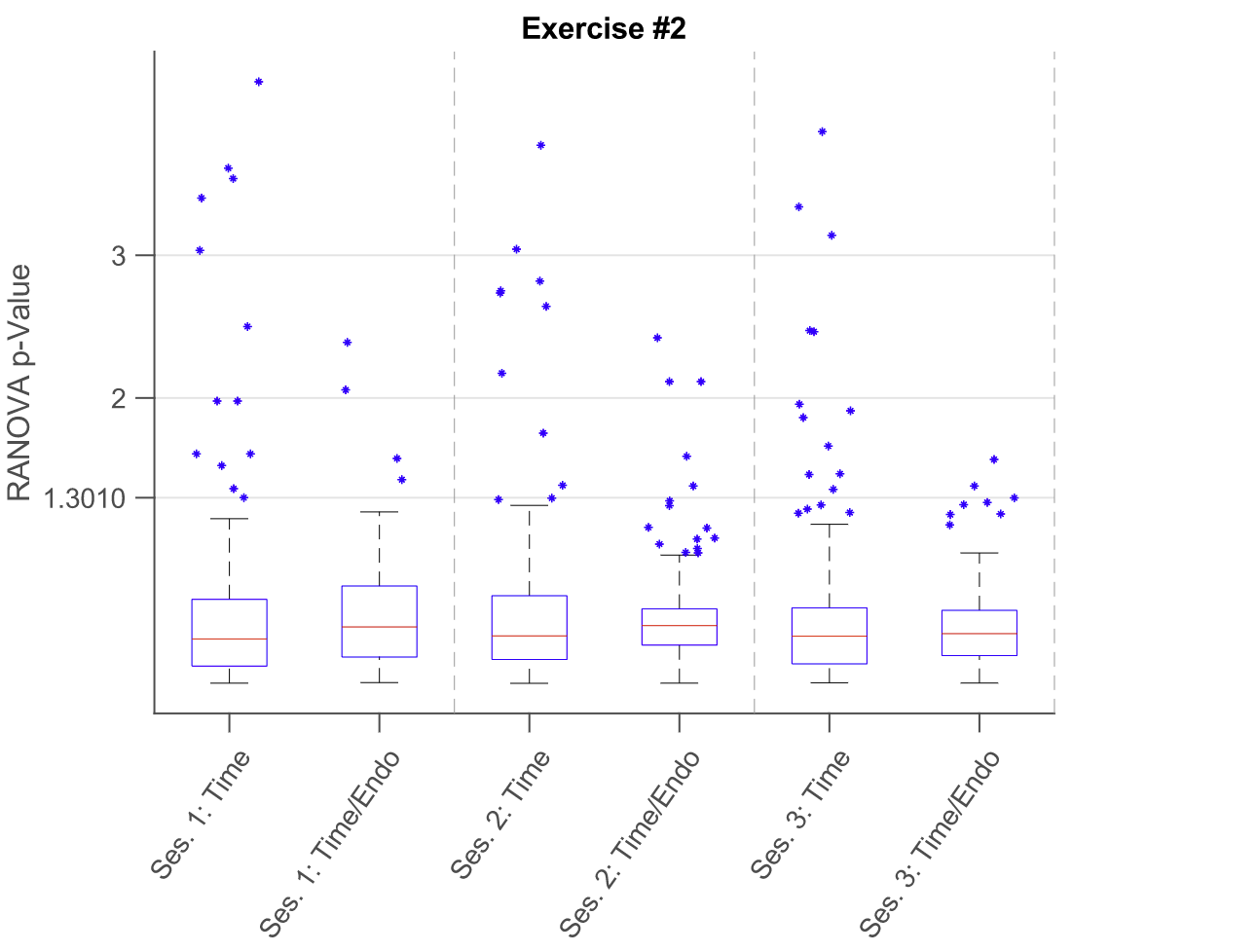


Fig. S2. Boxplots representing RANOVA p-Value calculation results for each session of exercise 2 converted to negative logarithmic (base 10) counter-part values, representing significance depending on time and the combination of time and the used endoscope type; ticks of the Y-axis mark the p-Value threshholds 0.05%, 0.01% and 0.001% (converted values being 1.3010, 2 and 3 respectively)


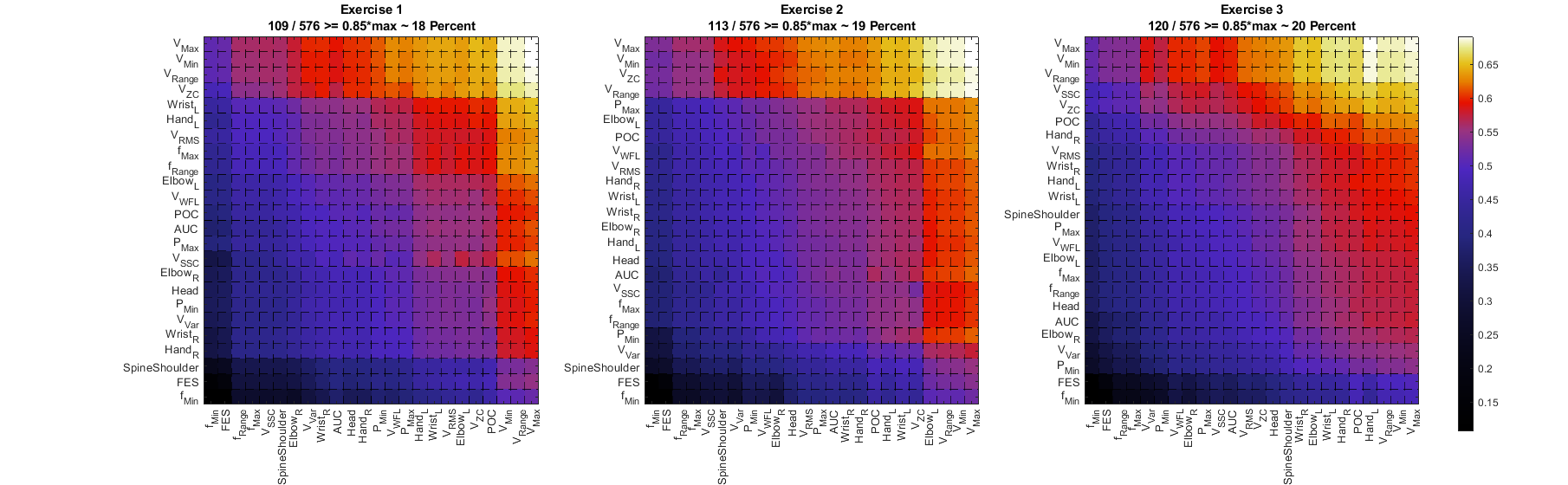
 Fig. S3. Overview of the heatmaps visualizing the averaged Euclidean distances between 2D values and 3D values of the features for each exercise dataset; Y-Axis contains the averaged 2D metric-specific feature vector which were used for element-wise comparison with the 3D metric-specific feature vector; each exercise dataset is designated in the title with an additional percentage calculation of features, that are within 15% of the highest average distance


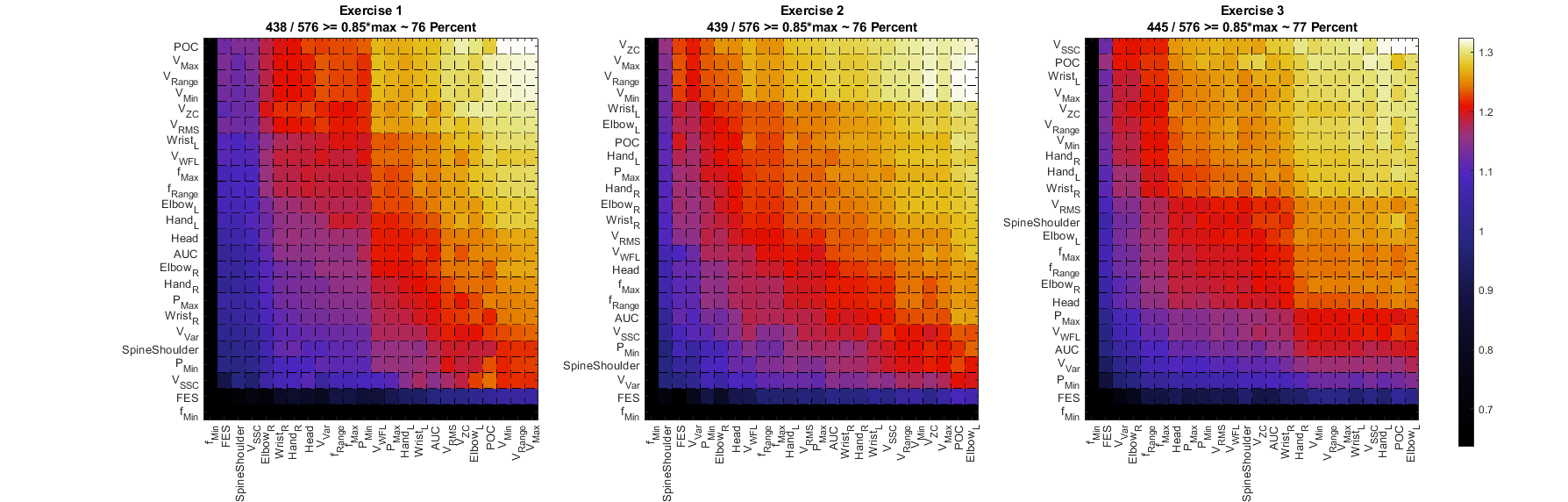
Fig. S4. Overview of the heatmaps visualizing the averaged Mahalanobis distances between 2D values and 3D values of the features for each exercise dataset; Y-Axis contains the averaged 2D metric-specific feature vector which were used for element-wise comparison with the 3D metric-specific feature vector; each exercise dataset is designated in the title with an additional percentage calculation of features, that are within 15% of the highest average distance

1. [↑](#endnote-ref-1)
